# Once a doctor, always a doctor

**DOI:** 10.1177/01410768231220312

**Published:** 2024-01-04

**Authors:** Connor S Qiu, Hutan Ashrafian

**Affiliations:** 1 School of Public Health, Imperial College London, London SW7 2AZ, UK; 2 Institute of Global Health Innovation, Imperial College London, London SW7 2AZ, UK

Historically, the first clinical signs were mentioned in a Sumerian text of Lugalbanda^1^ over 4000 years ago and an ‘art’ of medicine percolated to iteratively adopt empiricism over millennia. Hippocrates, who is widely attributed as the father of modern medicine, codified some of the domains that define what it means to be a medical doctor,^2^ although the concept of a doctor, someone who can diagnose and treat disease, predates this by many centuries. The idea that the sole purpose of medical school is to train medical doctors and that medical doctors practise clinical medicine became an accepted orthodoxy.^3^ Medical school and postgraduate education prepare individuals to practise medicine, and it does this reasonably well, but arguably, to improve retention and reduce attrition, it will likely need to modernise further and further emphasise formally building skills in innovation and creativity as a learnable and repeatable process. This might apply not only to the early stages of medical education, but also for the culture of medicine as a whole.^4^

There is perhaps a general underappreciation and underutilisation of the broader and holistic value of an education in medicine.^5^ This is perhaps most apparent in adjacent domains to healthcare in both the public and private sectors. The level to which this occurs varies internationally and there are cultural differences. Thus, there is an argument that medical education in the UK and globally, from medical school to specialty training, can further emphasise innovation and creativity in their curriculum. This will maximise the impact that medical students and the future practising physicians and surgeons in the UK will have on healthcare, maximising their societal value.

## What skills does medical education teach?

There is a unique thinking ability that is conferred throughout medical training, which when extrapolated can be particularly powerful and useful in all domains that involve complex problem solving. The ability to think laterally, to imagine all possibilities and to conceptualise the unmet need, is as important as a diagnostician on a medical ward as it is to solving some of the most complex challenges of our time ranging from climate change to geopolitical struggles.^6^

The trained ability to efficiently deal with high levels of uncertainty in the clinic room or the operating theatre with an organised stepwise approach, at all times of the day and night, under often enormous strains of total sleep deprivation and relentless task-switching is remarkable. This is a truly valuable commodity and transferrable skill. Despite increasing levels of burnout in physicians, as a population group, it is notable that the literature suggests doctors are more resilient than the working population as a whole.^7^

Beyond the obvious skills in communication, empathy and biomedical know-how, there is a curiosity and capability to learn complex technical concepts, which is universally shared among the cohort. There may be some concern about the ability of doctors to perform well in roles not directly related to patient care, but there are numerous examples in public and private life that counter this.

## How do doctors have societal impact in medical and other spheres?

The obvious way for doctors to impact upon health outcomes is to progress through specialty training, and perhaps eventually become leaders in their field, whether that be in diagnostic radiology or family practice. Familiar to those in academia or to those who once considered such a path, the structure of academic and medical departments is still traditional and pyramidical in structure.^8^ Training bottlenecks are topical. It is not for lack of ambition, hard work or talent, but it can involve a significant amount of luck to end up in a specialty of choice, not succumb to burnout and to reach the pinnacle of a respective field.^9^ There are also significant issues with retention across the medical profession. The recent NHS Workforce Long Term Workforce Plan does have some commendable ideas, but more needs to be done to practically encourage a more diverse pool of entrants into medical school to increase the quality of patient care, but also to defend against workforce attrition and enable a more dynamic, adaptable workforce that has a net positive impact on societal wellbeing.^10^

Some doctors will choose to expand upon their preparation or combine a past interest with their medical knowledge to make their unique contribution to the world. Hyphenated combinations, such as physician-economist,^11^ physician-politician,^12^ physician-engineer^13^ or physician-architect,^14^ may seem unworldly, but bold individuals with cross-disciplinary training, and perhaps bolder still, those that have left clinical practice entirely are transforming the landscape of completely different fields. The key to retention is to improve the innovation and creative preparation of doctors through medical school so that they can harness broad perspectives and see a wide range of possibilities to help them thrive in difficult patient care and work situations. In addition, this also improves the workforce’s general ability to contribute to complementary functions such as health policy or digital transformation. In addition, artificial intelligence is poised to revolutionise every facet of clinical medicine, shifting and changing every workforce job description.^15^ The physician-entrepreneur will play a key part in this.^16^ Promoting these educational measures will ensure that our future doctors will thrive in an ever-changing environment, continue in patient care and reduce burnout.

## What needs to be done?

Within the profession, attempts to introduce coercive policies to reduce attrition and facilitate retention in postgraduate medical education will likely not be highly successful. For example, policy changes should preserve the flexibility, autonomy and career capital of doctors to re-join and leave the profession as they wish. Employers and wider society should reframe the study of medicine into less of a vocational cause with a singular linear expectation, but as an excellent preparation for a variety of roles and futures with patient care as a core function. At the heart of this is emphasising the value of innovation and creativity in the medical school curriculum and postgraduate training. Similar comparisons can be drawn with other fields of study such as law, where the true value to society is perhaps behind training an individual’s ability to think, which incidentally in itself has parallels to engineering, and has a broader purpose than solely adding more lawyers to the workforce.^17^

We welcome a concerted and paradigm-shifting drive to substantially and efficiently increase the number of medical student places at higher institutions by governments around the world, including the UK,^18^ in a transformative capacity, not only to address the global workforce shortage. The addition of practised innovation and creativity to the medical curriculum can help overcome workforce attrition, and rather instil a skillset and perspective necessary to make the workforce resilient. Importantly, this will allow individual doctors to thrive in the challenging, but rewarding realities of modern medicine where future physicians will have the opportunities to generate value in medical and beyond-medical spheres.

## Declarations

### Guarantor

CSQ.

### Provenance

Not commissioned; editorial review.
